# Single‐Entry‐Point Thread Implantation Technique Using the Gerbera Pattern for Facial Skin Rejuvenation: A Prospective Intraindividual Controlled Clinical Study

**DOI:** 10.1111/jocd.70767

**Published:** 2026-02-19

**Authors:** Mai Huy Huân

**Affiliations:** ^1^ Hanoi Medical University Hanoi Vietnam

## Abstract

**Background:**

Mono and screw threads are increasingly used for facial skin rejuvenation. However, most currently described techniques rely on multiple skin entry points, which may increase procedural pain and negatively affect patient tolerability.

**Objective:**

To describe a single‐entry‐point thread implantation technique using the Gerbera pattern and to evaluate pain at needle penetration compared with a conventional multiple‐entry‐point technique in a prospective intraindividual controlled clinical study.

**Methods:**

This study included 20 adult female patients with mild‐to‐moderate facial skin aging. In paired facial regions (cheeks, malar areas, jawline, and temples), one side of the face was treated using the single‐entry‐point technique, while the contralateral side was treated using a conventional multiple‐entry‐point technique. Central regions (mid‐forehead and mid‐chin) were treated using shared modules due to anatomical considerations. Pain at needle penetration was assessed using a Visual Analogue Scale (VAS, 0–10).

**Results:**

All 20 patients completed the procedure on the side treated with the single‐entry‐point technique. Two patients discontinued the procedure early on the multiple‐entry‐point side due to pain but completed treatment on the single‐entry‐point side. Pain scores at needle penetration were markedly lower with the single‐entry‐point technique than with the multiple‐entry‐point technique. No serious adverse events were observed.

**Conclusion:**

The single‐entry‐point thread implantation technique using the Gerbera pattern significantly reduced pain at needle penetration compared with the conventional multiple‐entry‐point technique, demonstrating good tolerability in clinical practice.

## 1 Introduction

Thread implantation using mono and screw threads has become a common adjunctive procedure for facial skin rejuvenation, with the primary aim of improving skin quality through collagen stimulation and dermal remodeling [1, 2, 3].

In current clinical practice, most thread implantation techniques are based on multiple skin entry points, with each thread corresponding to a separate puncture site [2, 3]. Threads are typically arranged in linear, mesh‐like, or fan‐shaped patterns [2, 3]. As the number of implanted threads increases, the number of skin penetrations increases accordingly, potentially leading to greater procedural pain [4, 5].

From a patient perspective, pain at needle penetration is an important factor influencing acceptance of cosmetic procedure, particularly when repeat treatments are required [4, 5]. A review of publications indexed in PubMed and Scopus from 2010 to 2025 indicates that reported mono and screw thread techniques consistently employ a multiple‐entry‐point approach. Within this scope, no standardized technique using a single‐entry‐point to implant multiple threads as a repeatable module across the face has been described.

To address this limitation, a single‐entry‐point thread implantation technique using the Gerbera pattern was developed, in which multiple threads are inserted radially from a single skin entry point to reduce the number of punctures while maintaining appropriate thread distribution.

The objective of this study was to describe this technique and to evaluate pain at needle penetration in direct comparison with a conventional multiple‐entry‐point technique.

## 2 Materials and Methods

### 2.1 Study Design

This was a prospective intraindividual controlled clinical study, in which each patient served as her own control to minimize interindividual variability.

### 2.2 Patients

**FIGURE 1:**
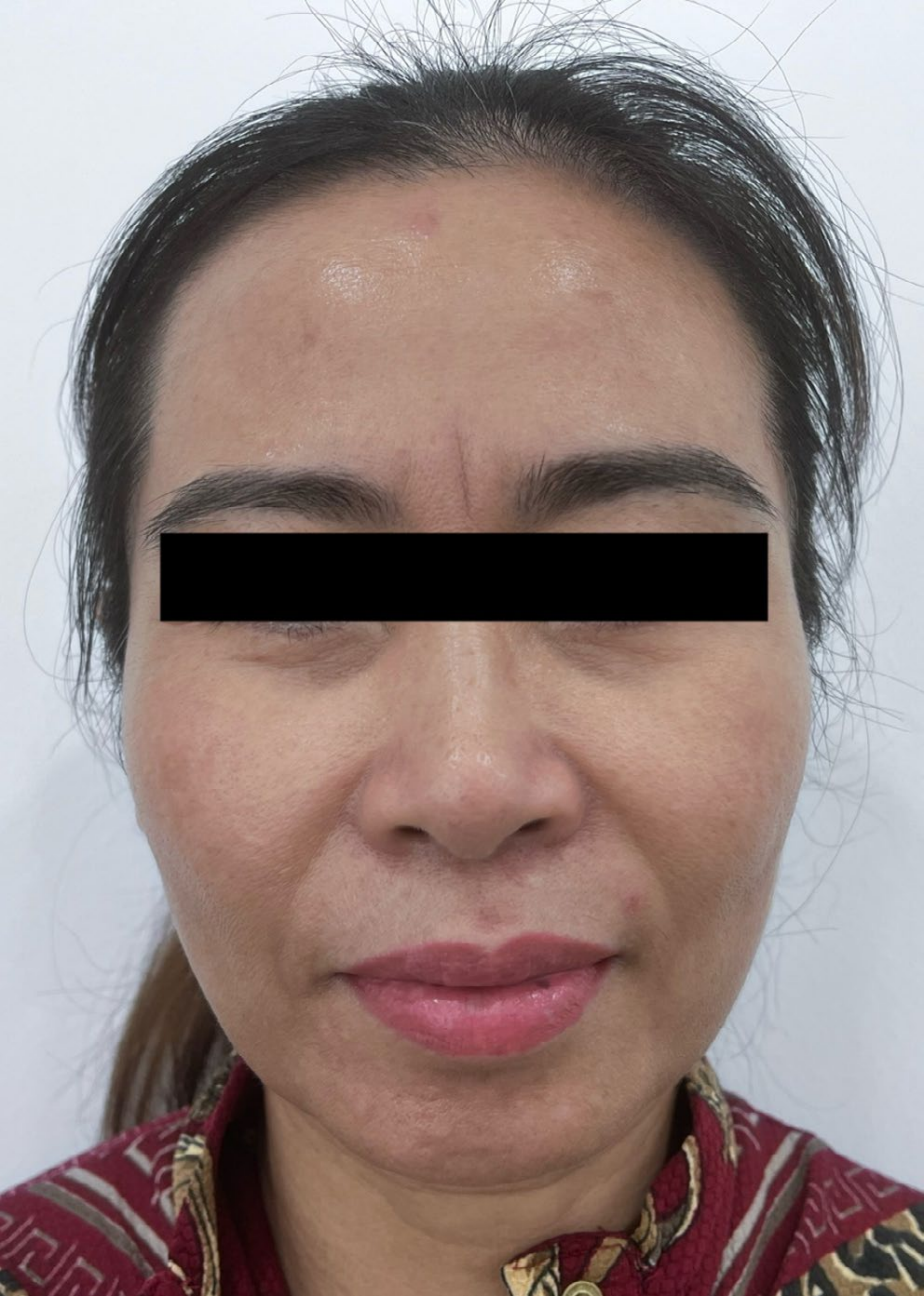
Before procedure. Frontal view of a 55‐year‐old female patient before the procedure.

Twenty adult female patients with mild‐to‐moderate facial skin aging were enrolled. Some patients were older than 50 years but did not exhibit severe soft‐tissue ptosis. Patients with severe facial ptosis, active skin infection, or a history of facial thread implantation within the previous 12 months were excluded. A representative baseline frontal view of a 55‐year‐old study participant is shown in Figure 1.

### 2.3 Treatment Areas and Intraindividual Comparison

**FIGURE 2:**
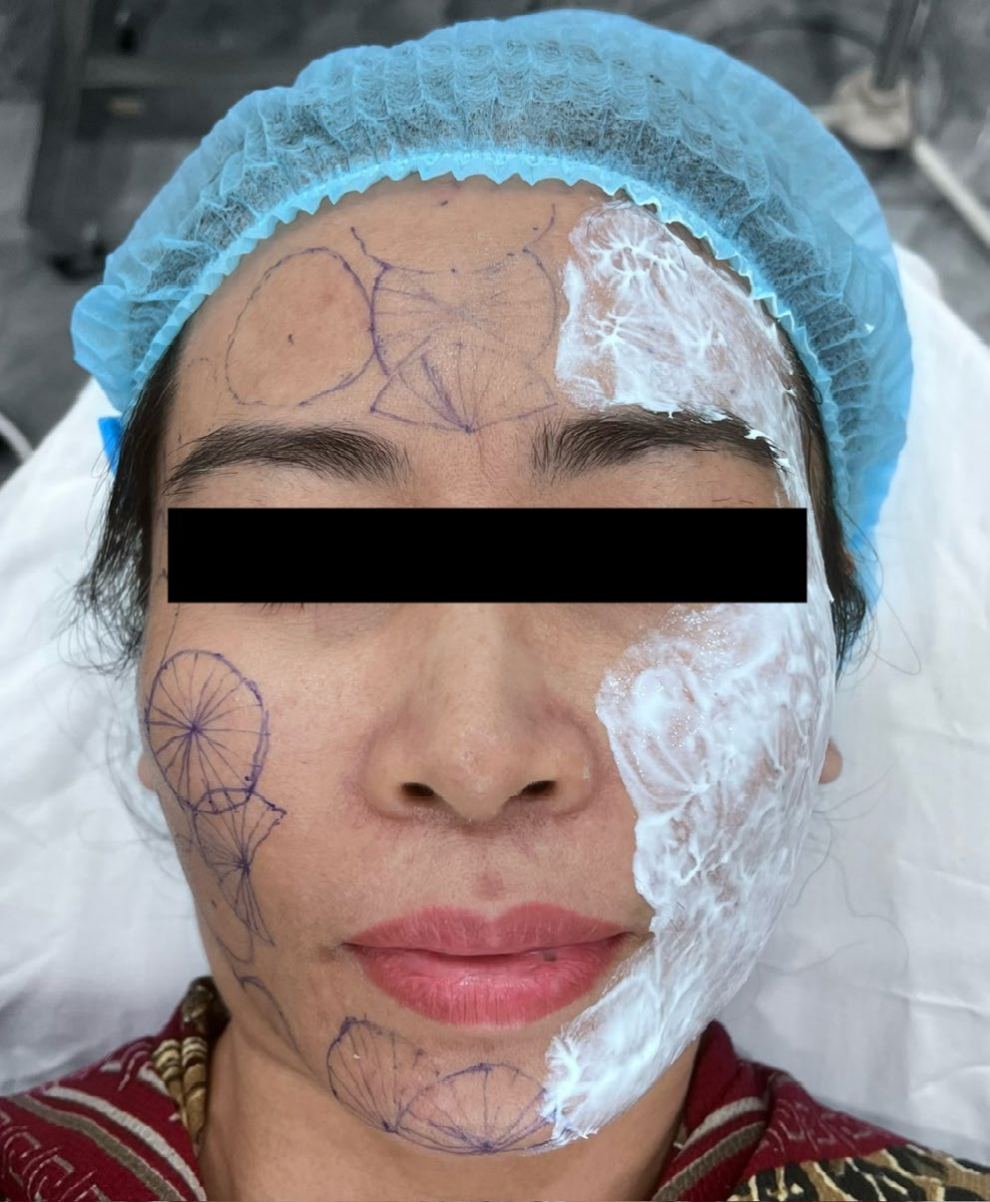
Treatment area mapping. Frontal view illustrating the designated treatment areas, including the forehead, temples, cheeks, malar areas, and jawline.

The cheeks, malar areas, jawline, and temples were treated separately on each side of the face to allow direct comparison between the two techniques. Central regions, including the mid‐forehead and mid‐chin, were treated using shared modules due to anatomical considerations. The designated treatment areas and facial mapping are illustrated in Figure 2.

### 2.4 Single‐Entry‐Point Mono Thread Implantation Technique Using the Gerbera Pattern

#### 2.4.1 Module Design Principle

**FIGURE 3:**
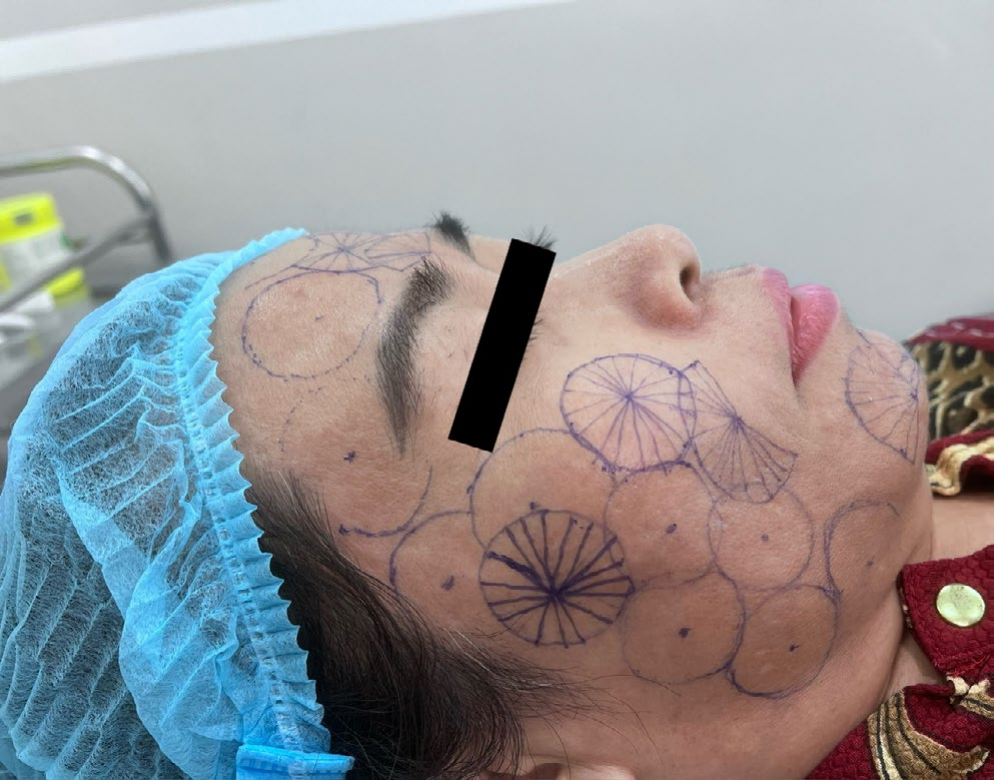
Gerbera pattern design. Schematic illustration of the Gerbera pattern. Each needle entry point serves as the center from which multiple threads are inserted radially, corresponding to the “petals” of the Gerbera pattern.

Thread implantation modules were designed to minimize the number of skin entry points while still achieving an appropriate thread distribution tailored to the anatomical characteristics and treatment objectives of each facial region. Each module corresponds to a circular radial unit centered on a single skin entry point. The schematic design of the Gerbera pattern module is shown in Figure 3.

#### 2.4.2 Preparation and Anesthesia

The treatment areas were cleansed and disinfected according to standard clinical procedures.

On the side treated with the single‐entry‐point technique, a small volume of local anesthetic (approximately 0.05 mL) was infiltrated subdermally at each needle entry point [6]. No anesthetic infiltration was performed along the needle pathway.

On the contralateral control side, anesthesia was achieved using topical anesthetic cream [6], in accordance with conventional thread implantation practice.

#### 2.4.3 Technique Execution

From each anesthetized entry point, a mono thread–loaded needle was introduced into the subdermal plane and advanced along a predefined vector. After the thread had been placed at the desired depth and length, the needle was completely withdrawn from the skin, leaving the thread in the intended position.

Subsequently, through the same skin entry point, another thread‐loaded needle was introduced along a different centrifugal vector. This sequence was repeated multiple times, with each insertion performed sequentially, to achieve an appropriate radial distribution of threads tailored to the anatomical characteristics and treatment objectives of each facial region.

**FIGURE 4:**
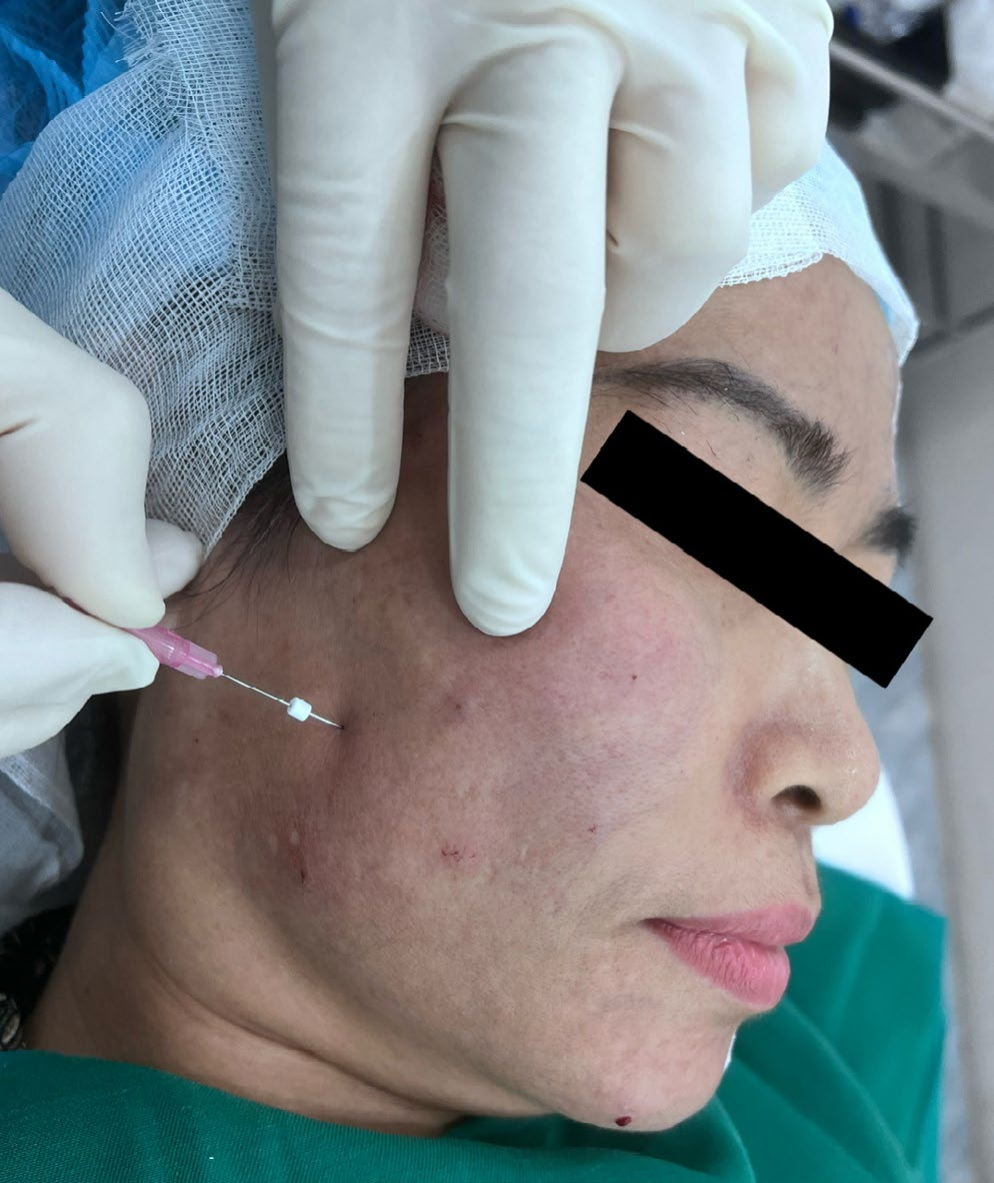
Single‐entry‐point technique (intraoperative). Intraoperative image demonstrating the single‐entry‐point thread implantation technique using the Gerbera pattern. Multiple threads are inserted in different directions through the same entry point.

This sequential single‐entry‐point maneuver is illustrated in the intraoperative photograph (Figure 4), demonstrating the use of a single skin entry point and the stepwise introduction of thread‐loaded needles. Because threads are inserted sequentially rather than simultaneously, the complete Gerbera pattern cannot be visualized at any single time point during the procedure.

**FIGURE 5:**
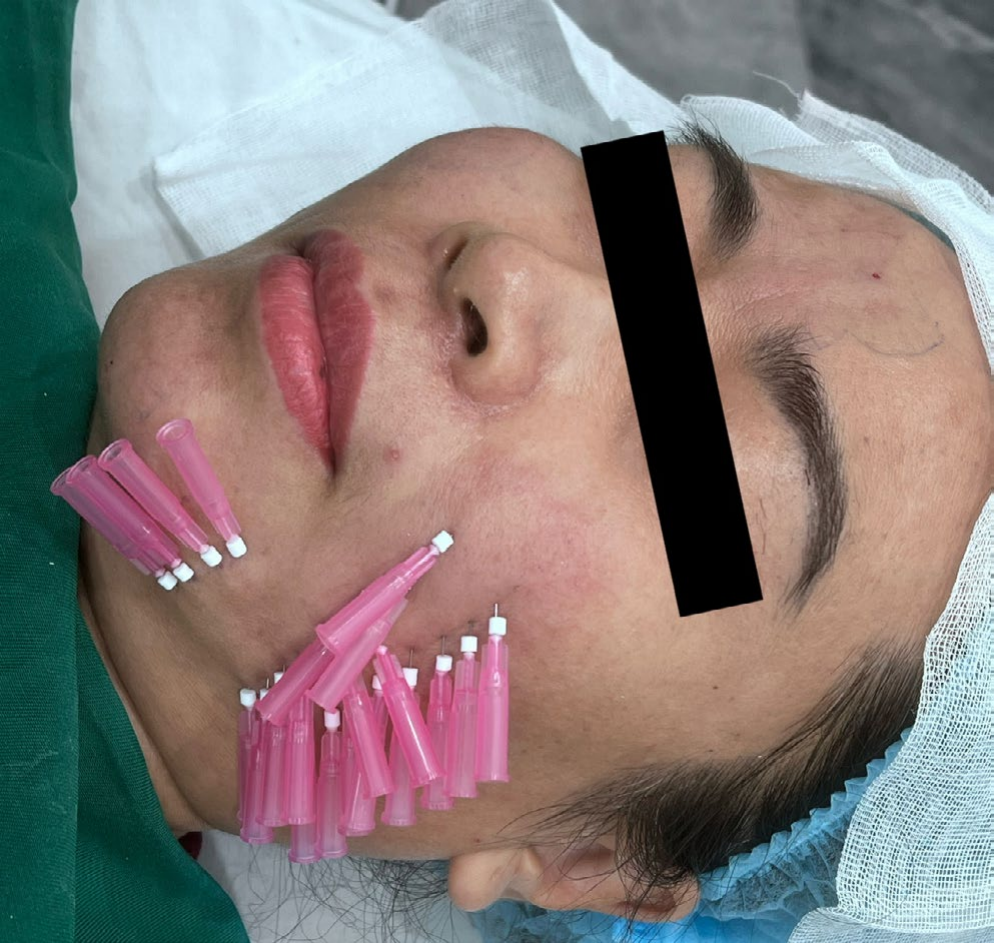
Multi‐entry‐point technique (intraoperative). Intraoperative image demonstrating the conventional multi‐entry‐point thread implantation technique.

In contrast to the single‐entry‐point technique, the conventional multi‐entry‐point approach involves inserting each thread through a separate skin puncture site along a predefined vector. Consequently, multiple independent entry points are required to achieve the desired thread distribution across the treated area. The intraoperative appearance of this conventional technique is presented in Figure 5.

### 2.5 Pain Assessment

Pain at needle penetration was evaluated using a Visual Analogue Scale (VAS) ranging from 0 (no pain) to 10 (worst imaginable pain).

### 2.6 Ethics Statement

The study was conducted in accordance with the ethical principles of the Declaration of Helsinki. Written informed consent was obtained from all participants prior to enrollment.

## 3 Results

**FIGURE 6:**
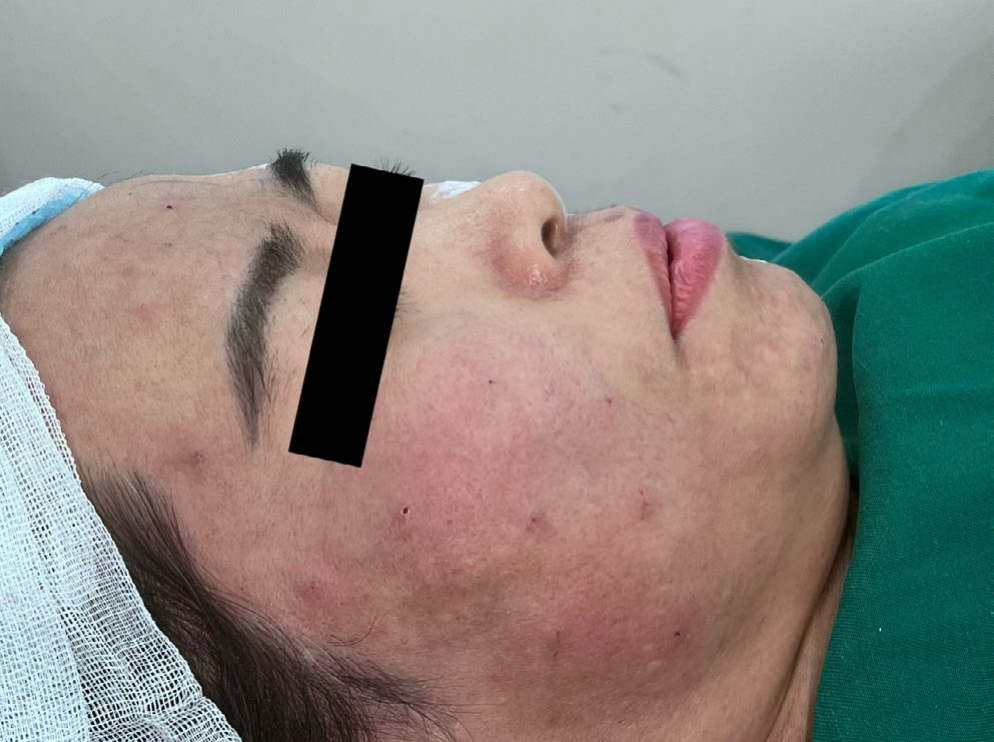
Post‐procedure image (Gerbera pattern technique). Image obtained immediately after completion of thread implantation using the Gerbera pattern technique.

**FIGURE 7:**
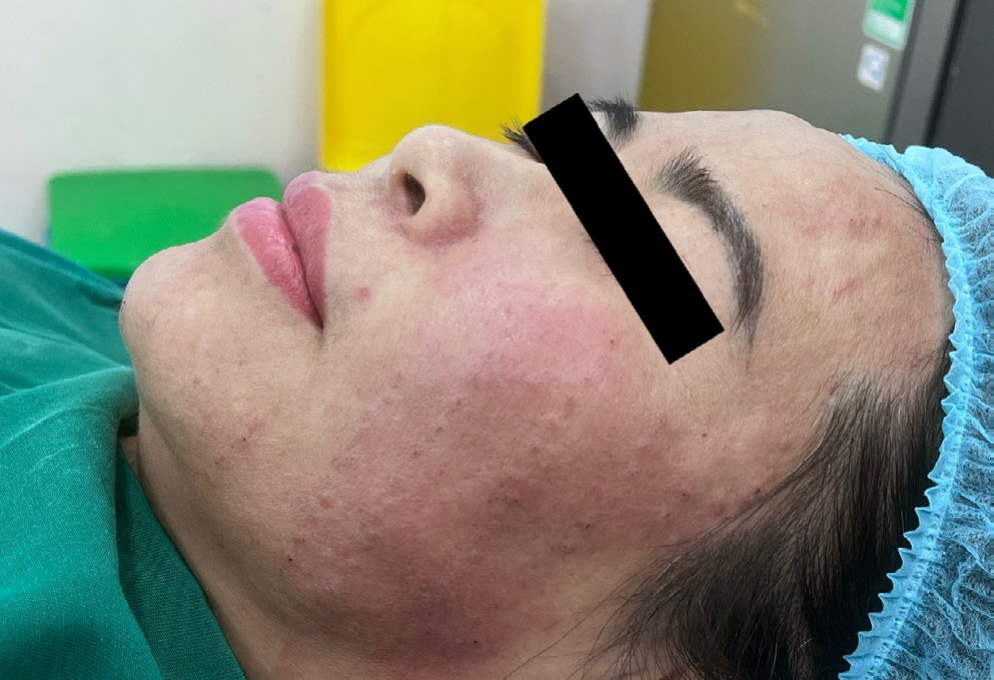
Post‐procedure image (multi‐entry‐point technique). Image obtained immediately after completion of thread implantation using the conventional multi‐entry‐point technique.

**FIGURE 8:**
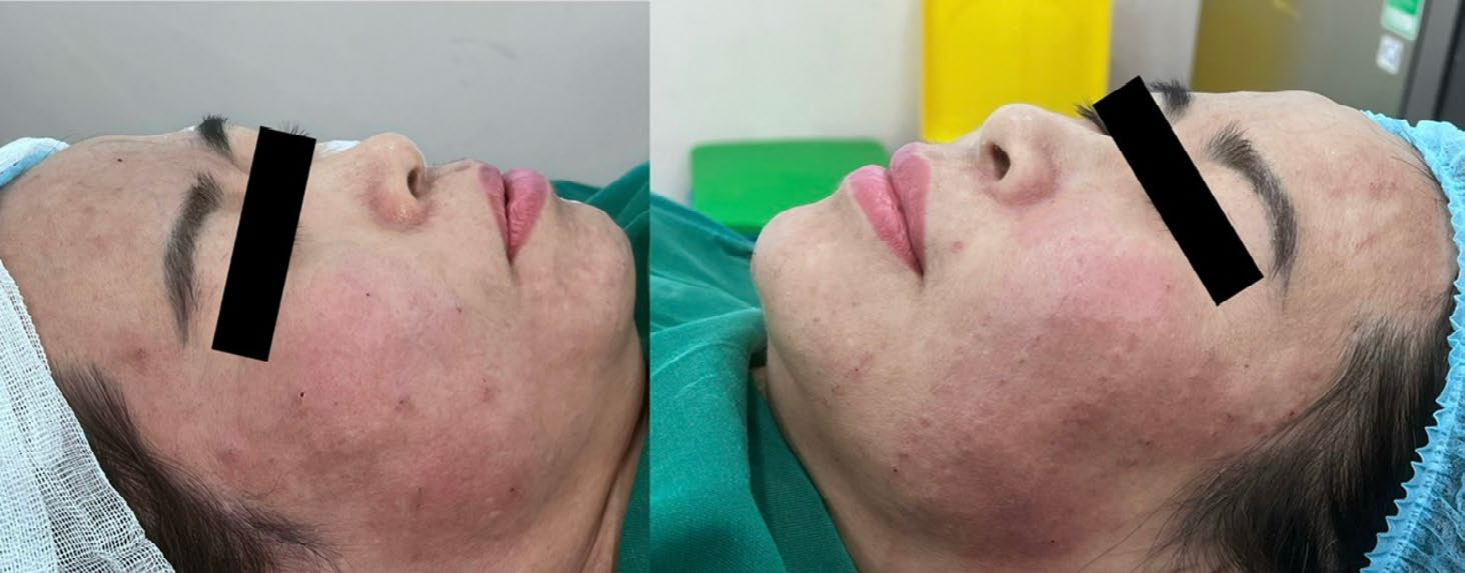
Comparison between techniques. Side‐by‐side comparison of the Gerbera pattern technique and the multi‐entry‐point technique immediately after the procedure.

**FIGURE 9:**
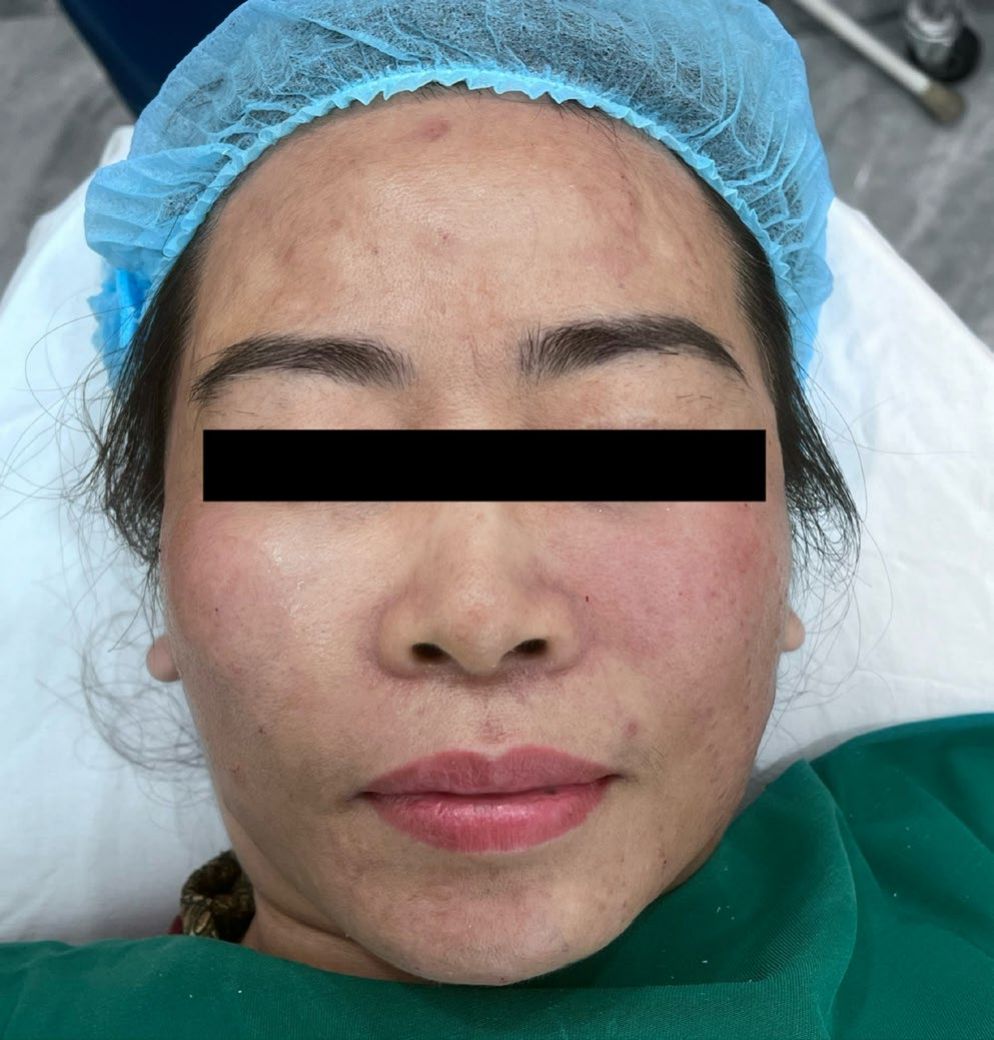
Post‐procedure frontal view. Frontal view obtained immediately after completion of the procedure on both sides of the face.

All 20 patients completed the procedure on the side treated with the single‐entry‐point technique. Two patients discontinued treatment early on the multiple‐entry‐point side due to pain during needle penetration but completed the full procedure on the single‐entry‐point side. Immediate post‐procedure appearance following the single‐entry‐point Gerbera technique is shown in Figure 6. The immediate post‐procedure appearance of the side treated with the conventional multi‐entry‐point technique is shown in Figure 7. A direct side‐by‐side comparison between the two techniques is presented in Figure 8. The final frontal view after the completion of treatment on both sides is presented in Figure 9.

Pain scores at needle penetration were markedly lower on the side treated with the single‐entry‐point technique than on that with the multiple‐entry‐point technique. Individual pain scores are summarized in Table 1. No serious adverse events were observed during the study.

**TABLE 1:** Pain at needle penetration (VAS 0–10).

Patient	Single‐entry‐point	Multiple‐entry‐point
Sm 01	0.6	5.4
Sm 02	0.8	5.7
Sm 03	0.5	5.2
Sm 04	0.7	5.9
Sm 05	0.9	Discontinued due to pain
Sm 06	0.8	Discontinued due to pain
Sm 07	0.6	5.5
Sm 08	0.7	5.8
Sm 09	0.5	5.3
Sm 10	0.6	5.6
Sm 11	0.8	5.9
Sm 12	0.7	5.4
Sm 13	0.6	5.5
Sm 14	0.9	6.0
Sm 15	0.7	5.6
Sm 16	0.6	5.3
Sm 17	0.5	5.2
Sm 18	0.7	5.7
Sm 19	0.8	5.9
Sm 20	0.6	5.4

*Note:* Two patients (Sm05 and Sm06) discontinued the procedure on the multiple‐entry‐point side due to pain but completed treatment on the single‐entry‐point side.

## 4 Discussion

This study demonstrates that the single‐entry‐point thread implantation technique using the Gerbera pattern is associated with good tolerability in clinical practice, as evidenced by lower pain at needle penetration than in the conventional multiple‐entry‐point technique.

The observed reduction in pain is likely attributable to a substantial decrease in the number of skin entry points when multiple threads are implanted through a single puncture, thereby reducing repeated stimulation of cutaneous nociceptors. In addition, the use of localized anesthesia with a small volume of anesthetic injected directly at the entry point allows effective pain control without the need for extensive subdermal infiltration.

From a practical standpoint, this characteristic may be particularly beneficial for patients with low pain tolerance or sensitive skin, who often have difficulty tolerating procedures involving multiple skin punctures. In these patients, reducing the number of entry points may not only decrease immediate procedural pain but also improve overall treatment experience, thereby enhancing acceptance of repeat procedures when indicated.

In contrast, conventional multiple‐entry‐point techniques often rely on topical anesthesia. While convenient and noninvasive, topical anesthesia may provide insufficient or inconsistent analgesia when numerous punctures are required. Wider subdermal anesthetic infiltration can improve pain control but increases procedural invasiveness and necessitates careful management of total anesthetic dosage.

This study has limitations, including a small sample size and the assessment of a single clinical outcome—pain at needle penetration. Nevertheless, this outcome directly reflects patient experience during the procedure and is highly relevant to patient‐centered care in facial skin rejuvenation.

## 5 Conclusion

The single‐entry‐point thread implantation technique using the Gerbera pattern reduced pain at needle penetration compared with the conventional multiple‐entry‐point technique in this intraindividual controlled study. The technique demonstrated good tolerability and may represent a suitable option in facial skin rejuvenation practice.

## Funding

The author has nothing to report.

## Ethics Statement

This was an independent, investigator‐initiated observational clinical study conducted in a private clinical practice setting. In accordance with local regulations and ethical standards applicable to private clinical practice–based observational research, formal institutional ethics committee approval was not required. The study was conducted in compliance with the ethical principles of the Declaration of Helsinki.

## Consent

Written informed consent was obtained from all patients for the publication of their clinical photographs and associated clinical information in this manuscript.

## Conflicts of Interest

The author declares no conflicts of interest.

## Data Availability

The data that support the findings of this study are available on request from the corresponding author. The data are not publicly available due to privacy or ethical restrictions.

## References

[1] A. Savoia , C. Accardo , F. Vannini , et al., “Autologous Fibroblast Stimulation by Polydioxanone Threads for Facial Rejuvenation,” Journal of Cosmetic Dermatology 13, no. 4 (2014): 302–307.

[2] J. De Benito , R. Pizzamiglio , and A. Cecchini , “Use of Absorbable Polydioxanone Threads for Skin Rejuvenation,” Journal of Cosmetic Dermatology 14, no. 2 (2015): 144–151.

[3] K. Y. Park , H. K. Kim , and B. J. Kim , “Smooth Thread Implantation for Facial Rejuvenation: Technique and Outcomes,” Journal of Cosmetic Dermatology 15, no. 4 (2016): 352–358.

[4] M. H. Gold , “Patient Comfort in Cosmetic Dermatologic Procedures,” Journal of Cosmetic Dermatology 12, no. 2 (2013): 103–109.23725303

[5] B. J. Kim , D. H. Lee , and M. N. Kim , “Assessment of Pain During Minimally Invasive Cosmetic Procedures,” Journal of Cosmetic Dermatology 17, no. 4 (2018): 601–607.

[6] M. Alam , J. S. Dover , and K. A. Arndt , “Anesthesia Techniques in Dermatologic Procedures,” Journal of the American Academy of Dermatology 47, no. 1 (2002): 120–126.

